# Indirect effect, through aspects of neighborhood affluence and racial/ethnic composition, of receiving a Section 8 voucher on the prevalence of psychiatric disorders among boys and girls in the Moving to Opportunity study

**DOI:** 10.21203/rs.3.rs-8263770/v1

**Published:** 2026-06-26

**Authors:** Anna Krasnova, Dustin T. Duncan, Jeremy Kane, Keely Cheslack-Postava, Sarah E. Tom, Kara E. Rudolph

**Affiliations:** UCSF: University of California San Francisco; Columbia University; Columbia University; Columbia University; Columbia University; Columbia University

## Abstract

The Moving to Opportunity (MTO) experiment randomized low-income predominantly Black and Hispanic families in high-poverty neighborhoods to a housing voucher, resulting in moves that changed neighborhood affluence and racial/ethnic composition. Unexpectedly, voucher receipt increased the risk of harmful mental health outcomes among boys but decreased it among girls. Black and Hispanic adolescents aged 10 to 19 years at final follow-up were selected. We estimated natural indirect effects of voucher receipt on past-year: 1) mood disorder, 2) externalizing disorder, and 3) post-traumatic stress disorder (PTSD), through mediators capturing the proportion of time spent in low-poverty census tracts, share of college graduates, Black, and Hispanic residents, stratified by sex. We used a nonparametric, multiply robust estimator. Voucher receipt increased time living in more affluent and slightly less segregated neighborhoods, which was protective against externalizing disorders among boys and girls, and mood disorders among girls. However, among boys, these neighborhood changes explained most of the harmful effect of voucher receipt on mood disorders. We estimated null indirect effects on PTSD among boys and girls. These findings indicate that the mediated effects of voucher receipt through neighborhood poverty and racial/ethnic composition vary by mental health outcome and sex.

## Introduction

Observational studies show a consistent association between residing in high-poverty neighborhoods and a higher prevalence of psychiatric disorders in children and adolescents, even after controlling for individual-level poverty and other confounders.^1–8^ Neighborhood poverty, a fundamental cause, limits access to physical, financial, environmental, and social resources that aid individuals in avoiding diseases and their consequences.^9,10^ It affects all residents but is especially harmful to the mental health of children and adolescents,^4,5,11^ whose brains are rapidly developing.^12,13^ However, the association between neighborhood poverty and child and adolescent psychiatric disorders may be a byproduct of unmeasured confounding by family characteristics.^5^

One way to minimize unobserved confounding is through experimental design,^14^ but exposure to residing in a poor neighborhood typically cannot be randomized. However, the Moving to Opportunity (MTO) housing experiment, described in detail elsewhere,^15,16^ randomly assigned low-income families in five major U.S. cities to a low-poverty voucher, a traditional voucher, or an in-place control group.^15,16^ Families in the low-poverty voucher group were offered a voucher restricted to census tracts with less than 20% poverty, offered assistance in their housing search, and required to stay for at least one year in the new neighborhood.^15,17,18^ Traditional voucher families could use their vouchers without geographical restrictions and without special assistance.^15,17,18^ Among low-poverty voucher and traditional voucher families, 48% and 63%, respectively, relocated with a voucher.^17^

In terms of long-term mental health outcomes, 10 to 15 years after random assignment, voucher receipt was generally beneficial for adolescent girls but harmful for adolescent boys.^15,19^ This was true for internalizing disorders, characterized by disordered mood, and externalizing disorders, whose key feature is dysregulated behavior.^15,19,20^ Specifically, voucher receipt had no effect or lowered the prevalence of internalizing and externalizing disorders among adolescent girls and had no effect or increased the prevalence of these outcomes among boys.^16^

Voucher receipt, through moving, altered neighborhood affluence and racial/ethnic composition.^15^ After the initial lease-up (e.g., finding and leasing a private-market unit with a Section 8 voucher^15^), voucher recipient families tended to move to poorer neighborhoods, which diluted the beneficial effect of residing in a low-poverty neighborhood, and exposed adolescents to harmful exposures including violence, and low-quality housing. Two-thirds of the control group also moved since baseline, often to more affluent neighborhoods, lowering their exposure to high-poverty neighborhoods.^15^ Voucher receipt also affected the racial/ethnic neighborhood composition. Through moving, voucher receipt slightly reduced the concentration of Black and Hispanic residents, which is indicative of reduced racial/ethnic segregation, a change that some hypothesized would be beneficial for mental health.^22^ However, moving to neighborhoods with fewer Black and Hispanic residents could have increased participants’ exposure to racial/ethnic discrimination, which is harmful to mental health.^23^ At baseline, MTO families resided in neighborhoods with 80% or more racial/ethnic minorities.^15^ In contrast, 5–7 years post-randomization, low-poverty voucher recipients who moved resided in neighborhoods wherein 51% of residents were racial/ethnic minorities.^15^

Voucher receipt, through moving, altered various contexts important for adolescent mental health, including neighborhood affluence and racial/ethnic composition.^15^ We hypothesized that these changes could partially explain MTO's unexpected findings. We further hypothesized that sex is a moderator that affected the strength and/or direction of these mediating mechanisms. In this paper, we estimated the indirect pathway, through racial/ethnic composition and aspects of neighborhood affluence, linking voucher receipt to mental health outcomes in adolescents, stratifying by sex.

## Methods

### Data Source and Sample Selection

We utilized the MTO dataset (1994–2010), described in detail previously.^15,16^ We limited the sample to ages 10 to 19 years at the final follow-up in agreement with how the World Health Organization defines adolescence,^21^ and because mental health modules were only administered to those 20 years and younger.^16^

We excluded adolescents from the Baltimore site (n = 450), as has been done in prior studies,^24,25^ because at this site receipt of a voucher was not associated with a subsequent move. We also excluded adolescents whose race and ethnicity were not non-Hispanic Black or Hispanic (n = 200). The population we are making inferences about consisted of non-Hispanic Black or Hispanic adolescents in the MTO, excluding the Baltimore study site. In the sample that met these criteria, we excluded boys and girls with missing values in mediators and outcomes (n < 15). The rounded study sample consisted of 3300 adolescents. All values have been rounded in accordance with the Census regulations and analyses were done in R version 4.2.2. Columbia University Institutional Review Board determined that this study was nonhuman subjects research.

#### Treatment Assignment

Randomly assigned voucher receipt (i.e., any voucher or no voucher) in 1994–1998 is the treatment of interest. We combined two voucher treatments as has also been done in prior studies to increase power and improve model parsimony.^25–28^

#### Mental Health Outcomes

We examined three binary (yes/no) self-reported outcomes measured at the final assessment (2008–2010): 1) any past-year diagnostic statistical manual, fourth edition (DSM-IV) mood disorder (depression, bipolar I/II/subthreshold, and mania/hypomania/hypomania subthreshold), 2) externalizing disorder (intermittent explosive disorder, conduct disorder, and oppositional-defiant disorder), and 3) post-traumatic stress disorder (PTSD).^16^ In a sensitivity analysis, we also examined major depressive disorder (MDD) by itself. These DSM-IV psychiatric disorders were assessed by interviewing adolescents via the Composite International Diagnostic Interview (CIDI),^29^ which has high reliability and validity for mental health outcomes.^30,31^

#### Mediators

We examined a bundle of four duration-weighted census tract mediators over the 10 to 15-year study period that capture key aspects of neighborhood affluence and racial/ethnic composition. These included: 1) proportion of time spent in a census tract with less than 20% poverty (i.e., the duration of family’s residence in such a census tract^16^), 2) share of college graduates, 3) proportion of Black residents, and 4) proportion of Hispanic residents. Additionally, we conducted a sensitivity analysis with the number of moves as an additional mediator.

#### Moderators

Self-reported biological sex is hypothesized to act as a moderator of the associations between mediators and mental health outcomes.

### Baseline Covariates

We adjusted for site because random voucher assignment was conditional on it.^32^ We also adjusted for the other baseline covariates which were confounders of the mediator-to-outcome relationship. Specifically, we adjusted for child’s baseline age, race/ethnicity, behavioral/emotional problems, gifted status, and household size (Table 1). We adjusted for primary adult caretaker’s education level (graduated high school or obtained graduate equivalency diploma vs. not), working for pay, marital status (never married vs. not), birth of a child before 18 years old, reasons for moving with a voucher, receipt of Aid to Families with Dependent Children (AFDC), prior Section 8 voucher receipt, and baseline neighborhood poverty percentage.

#### Post-treatment Intermediate Confounders of the Mediator-Outcome Relationship

We adjusted for a post-treatment intermediate confounder - moving with the voucher - measured within 90 days of voucher receipt. This variable is influenced by the exposure and, in turn, has the potential to affect both the mediators and the outcomes.^33^

##### Notation

We let *A* represent the treatment assignment, *Y* the outcome, *M* the hypothesized mediator or the bundle of mediators, *W* the vector of measured baseline covariates, *Z* the measured post-treatment intermediate confounder, and *V* the strata (e.g., boys or girls).^34^ In addition, we let *Y*_*a*_ represent a potential outcome, and *E*[*Y*_*a*_] the expected mean potential outcome if all subjects were exposed to *A* = *a*.^35^ Furthermore, *M*_*a*_ refers to the potential mediator if we set *A* = *a*, and *Y*_*a,m*_ to the potential outcome if we set the value of exposure *A* to *a* and the value of mediator *M* to *m*.^28,36^

#### Causal Estimands

First, we estimated the effect of receiving a voucher on each mental health outcome for boys and, separately, girls, adjusted for study site. Second, we estimated the effect of voucher receipt on each hypothesized mediator, adjusted for study site. Third, we estimated the natural direct effect (NDE), which is the difference in the following expected values of the outcome: 1) if an individual received a voucher and had the mediator bundle value they would have had if they did not receive a voucher, and 2) if an individual did not receive a voucher and had the mediator bundle value they would have had under these conditions.^36,37^ Using notation, it is $\left[Y_{1,M_{0}}-Y_{0,M_{0}}\right]$.^36^ Finally, we estimated the natural indirect effect (NIE) of receiving a voucher on the prevalence of mental health outcomes through the bundle of mediators that included markers of neighborhood affluence and racial and ethnic composition. In a sensitivity analysis, we added the number of moves to the bundle of mediators. The NIE is the average difference between the following potential outcomes: 1) an individual received a voucher and had the mediator bundle values they would have under these conditions, and 2) an individual received a voucher but had the values of the mediator bundle they would have had if they did not receive a voucher. Using notation, NIE can be expressed as $\left[Y_{1,M_{1}}-Y_{1,M_{0}}\right]$.

### Assess Identifiability

To identify natural direct and indirect effects from observed data, the following assumptions must be met: 1) no unmeasured confounding between treatment *A* and outcome *Y*,^36,38^ 2) no unmeasured confounding between mediator *M* and outcome *Y*,^36,38^ 3) no unmeasured confounding between treatment *A* and mediator *M*, 4) monotonicity between treatment and intermediate confounder (i.e., no families actively resisted the intervention^39,40^), 5) positivity of treatment condition *a*, 6) positivity of mediator value *m*, 7) consistency, and 8) no interference between the units. These assumptions are described in detail in eAppendix A.

### Choose and Apply Estimator

We utilized logistic regression adjusted for study site to estimate the effect of voucher receipt on each outcome and each mediator. We then used a recently developed nonparametric, multiply robust estimator^40^ to estimate the NIE of voucher receipt on the mental health outcomes through the bundle of mediators (e.g., aspects of neighborhood affluence and racial/ethnic composition), under the above-mentioned identifiability assumptions.^36^ This estimator can accommodate multiple mediators.^36^

### Missing Data

We utilized multiple imputation by chained equations (MICE) to impute missing covariates to minimize potential selection bias.^41^ We included all variables present in subsequent analyses, as well as variables that may improve prediction accuracy.^41^ We generated 10 imputed datasets, performed analyses on each dataset, and used Rubin’s rules^42^ to combine estimates and standard errors.

Fewer than 15 individuals were missing mediators or outcomes. We used MTO weights to minimize bias associated with differential loss-to-follow-up over the 10 to 15 years.^16^

## Results

### Descriptive Analysis

The rounded sample included 3300 non-Hispanic Black and Hispanic adolescents 10 to 19 years old at the final evaluation, recruited from Boston (18%), Chicago (29%), Los Angeles (28%), and New York (25%). Table 1 summarizes survey-weighted baseline characteristics. The mean baseline age was 3.82 years (standard deviation [SD] = 0.04). Most adolescents were non-Hispanic Black (66%) and approximately half were boys (49%). Mean baseline poverty was 0.55 (SD = 0.00), 82% of participants’ families received AFDC, 36% received a Section 8 voucher before, and 11% moved 3 or more times previously.

### Effect of Voucher Receipt on Mediators and Outcomes

Estimated effects of voucher receipt on mediators and outcomes, adjusted for study site, is shown in Table 2. Among boys and, separately, girls voucher receipt had a small to medium estimated effect on measures of neighborhood affluence and racial/ethnic composition. For example, voucher receipt led to a 0.20 (95% CI: 0.15, 0.25) and 0.24 (95% CI: 0.20, 0.29) estimated increase in duration-weighted time spent in neighborhoods with less than 20% poverty among boys and girls, respectively. It also led to a small increase in the duration-weighted proportion of the share of college graduates and the proportion of Black and Hispanic neighborhood residents among boys and girls. Finally, voucher receipt also led to an increased number of moves (RD = 0.63, 95% CI: 0.43, 0.84 among boys, RD = 0.62, 95% CI: 0.43, 0.82 among girls).

Among boys, voucher receipt led to a slightly greater risk of any past-year DSM-IV mood disorder (RD = 0.01, 95% CI: −0.02, 0.04), any externalizing disorder (RD = 0.02, 95% CI: −0.02, 0.06), and PTSD (RD = 0.02, 95% CI: 0.01, 0.04), but had no effect on MDD (RD = 0.00, 95% CI: −0.03, 0.02). Among girls, voucher receipt led to a reduction in the risk of these outcomes. Specifically, a reduction in risk of any past-year mood disorder (RD = −0.04, 95% CI: −0.08, 0.00), externalizing disorder (RD = −0.03, 95% CI: −0.07, 0.02), PTSD (RD = −0.01, 95% CI: −0.04, 0.01), and MDD (RD = −0.02, 95% CI: −0.05, 0.01).

### Natural direct and indirect effects

Table 3 shows the estimated direct and indirect effects of voucher receipt on each mental health outcome stratified by sex. Among boys, the majority of the voucher receipt effect (RD = 0.02, 95% CI: 0.00, 0.03) on the increased risk of mood disorder including MDD (RD = 0.01, 95% CI: 0.00, 0.02) operated through the bundle of mediators (i.e., greater time spent in more affluent and slightly less segregated neighborhoods), which was replicated in the sensitivity analysis. In contrast, among boys, there was an estimated protective effect (RD = −0.03, 95% CI: −0.09, 0.03) of voucher receipt, through the bundle of mediators, on externalizing disorders. Lastly, there was an estimated null indirect effect of voucher receipt on PTSD (RD = 0.00, 95% CI: −0.02, 0.02).

Among girls, a consistent pattern was observed for the effect of voucher receipt on the outcomes of mood disorder, MDD, and externalizing disorder. Compared to no voucher, part of the protective effect of voucher receipt on mood disorder (RD = −0.01, 95%: −0.04, 0.02), MDD (RD = −0.01, 95% CI: −0.03, 0.01), there was an estimated protective effect (RD = −0.03, 95% CI: −0.09, 0.03) of voucher receipt, through the bundle of mediators, on externalizing disorders. Lastly, there was an estimated null indirect effect of voucher receipt on PTSD (RD = 0.00, 95% CI: −0.02, 0.02).

In a sensitivity analysis, we added the number of moves to the bundle of mediators (eAppendix B). This had minimal effect on our estimates

## Discussion

Housing voucher receipt was estimated to have an adverse effect on boys’ mood disorders, externalizing disorders, and PTSD, while demonstrating a protective effect on these outcomes among girls, consistent with prior research.^16,19,28^ Among boys, the adverse effect of voucher receipt on mood disorders was partially mediated by relocation to more affluent neighborhoods with slightly lower proportions of Black and Hispanic residents and a slightly higher proportion of college graduates. However, voucher receipt had an indirect protective effect on externalizing disorders through this same set of neighborhood characteristics. Among girls, part of the beneficial effect of voucher receipt on mood and externalizing disorders was mediated by changes in neighborhood affluence and racial/ethnic composition. There was an estimated null indirect effect of voucher receipt on PTSD for either boys or girls.

According to the U.S. Department of Commerce, high-poverty neighborhoods have 20% or more residents living in poverty.^43^ While, voucher receipt led to an overall decrease in neighborhood poverty, it was often not substantial and most families remained living in high-poverty neighborhoods.^15^ That said, voucher receipt slightly increased the proportion of time spent in non-poor, slightly less racially/ethnically segregated neighborhoods, and we estimated that these modest changes contributed to the overall effects of voucher receipt on all outcomes, except PTSD.

For externalizing disorders, living in slightly more affluent and slightly less racially/ethnically segregated neighborhoods resulting from voucher receipt was modestly beneficial for both boys and girls. One possible reason is that increased affluence and decreased racial/ethnic segregation could have changed social norms, improved neighborhood safety, and increased access to resources, factors beneficial for externalizing disorders.^44–46^ The overall harmful effect of voucher receipt on externalizing disorders among boys could have occurred through other pathways, including residential instability and interaction with risk-taking peers, which increased as a result of voucher receipt^25,28^ and have been found more harmful to the mental health of boys than that of girls.^25,28,47,48^ Residential instability may have disrupted social networks harming the mental health of children and adolescents.^49,50^ Boys from voucher recipient families were also more likely to form friendships with peers who used drugs.^23, 25,48^ These and other factors resulting from voucher receipt may have outweighed the benefits of residing in slightly more affluent and less segregated neighborhoods.

For mood disorders, living in more affluent and slightly less racially/ethnically segregated neighborhoods as a result of voucher receipt was slightly beneficial for girls but harmful for boys. One possible reason for this is that boys may be more sensitive than girls to differences in affluence between themselves and their neighbors.^51^ In addition, moving to neighborhoods with fewer Black and Hispanic residents among voucher recipients could have increased exposure to racial and/or ethnic discrimination among Black and Hispanic adolescents, especially boys.^52–55^ Hence, boys may have had a higher risk of mood disorders due to feelings of inadequacy and socioeconomic and racial/ethnic discrimination. In contrast, girls may have been better equipped than boys to adjust to residential instability, including school changes and disruption in social networks^23,56^ due to differences in parental socialization.^57^ In addition, girls may have benefited from voucher receipt through multiple pathways, including a reduction in sexual harassment.^23^

For PTSD, living in more affluent and slightly less racially/ethnically segregated neighborhoods resulting from voucher receipt had no effect among boys or girls. One possible reason is that our study was underpowered to detect indirect effects of voucher receipt on PTSD, which was less prevalent than larger categories of mood and externalizing disorders that included multiple diagnoses. In addition, MTO was underpowered to detect the indirect effects of the two vouchers, considered separately, on adolescent mental health outcomes. Since the risk of PTSD was especially high in the low-poverty group compared to the control group, combining the voucher groups could have diluted the indirect effect through aspects of neighborhood environment.^19^ In addition, changes in other aspects of neighborhoods that were not captured by our mediators, such as traumatic events as a result of friendships with risk-taking peers, could have been responsible for the increased risk of PTSD.

This study has several limitations. MTO did not collect measures of social class discrimination, a hypothesized pathway for the unexpected harmful effects^23^ among boys whose families moved with a voucher to more affluent neighborhoods. We were unable to evaluate this pathway or to test how the combination of neighborhood poverty and class discrimination affects adolescent mental health.^58^ In addition, some mediators of interest, such as perceived racial/ethnic discrimination, could not have been examined in this study because they were only measured for a subset of included children, who were 12 years and older at the interim follow-up. Furthermore, we employed MICE to minimize possible bias due to missing values in covariates. However, we cannot rule out a missing-not-at-random mechanism.

Measures of racial/ethnic segregation available in the MTO were imperfect. At baseline, neighborhoods were highly segregated (> 80% racial/ethnic minority).^15^ Therefore, we used a decrease in the percentage of Black and Hispanic residents as a proxy for reduced segregation. In addition, we evaluated mediators as a bundle because affluence is highly correlated with racial/ethnic composition in the U.S. due to the legacy of racist policies such as redlining.^59–61^ Therefore, we were unable to identify if aspects of neighborhood affluence or racial/ethnic composition were driving the estimated indirect effects. In addition, we estimated intention-to-treat effects because evaluating the effect of voucher receipt is valuable for housing policy, which can assign vouchers but can’t force families to utilize them. Per-protocol effects, which represent moving with the voucher, are also policy-relevant. They were not estimated in this study and are an important direction for future studies. Furthermore, MTO findings may not generalize to all Section 8 eligible families because participants were already receiving some public subsidies and on the voucher waiting list, indicating they were highly motivated and able to qualify, unlike many families in need of such assistance.^62^

In a sensitivity analysis, we considered the number of moves as an additional potential mediator. Frequent moves may harm child and adolescent mental health.^25,28,49,50^ Ignoring this potential mediator may have been a source of bias, but we found that incorporating it had little effect on indirect effects.

We could have estimated interventional direct (IDE) and indirect effects (IIE) instead of natural ones because the identifiability assumptions are more likely to hold for the former. Specifically, point identification of the NIE generally requires the assumption of no measured or unmeasured post-treatment confounding between the mediator and outcome, while the IIE does not have this identifiability assumption.^36^ There are several measured post-treatment confounders that may be relevant for our research question, including moving with a voucher, residential instability, and risk-taking peers.^15^ We adjusted for moving with a voucher by using an alternative identification result for the NIE^36,40^ that allows for adjustment of a post-treatment confounder assuming a monotonic relationship between it and treatment. We also included the number of moves in the bundle of mediators, but we did not adjust for other measured post-treatment confounders. A future study could estimate the IIE and compare the results to the estimates in this study. We decided against estimating the IIE because of its limitations, such as not being easily interpretable and not decomposing the average treatment effects.^36^ In contrast, natural effects are easily interpretable (“descriptive”) and can be decomposed into direct and indirect components.^36^

## Supplementary Material

Supplementary Files

This is a list of supplementary files associated with this preprint. Click to download.

- eAppendix.docx

## Figures and Tables

**Table 1:** Characteristics of adolescents, 10 to 19 years old, enrolled in the MTO in Boston, Chicago, New York, and Los Angeles (1994–2010) stratified by random voucher assignment and sex^a^ (n = 3300^b^).

Characteristic	All	Boys		Girls		
		Control	Voucher	Control	Voucher	Missing
Boston (%)	18	18	17	19	19	0
Chicago (%)	29	29	29	26	29	
Los Angeles (%)	28	26	30	27	28	
New York (%)	25	26	24	27	25	
**Child's characteristics**						
Age, mean (SD)	3.82 (0.04)	3.75 (0.11)	3.86 (0.07)	3.88 (0.10)	3.80 (0.07)	0
Boys (%)	49					
Black non-Hispanic (%)	66	67	67	65	65	0
Behavioral/emotional problems (%)	1	1	1	1	1	0
Gifted (%)	3	3	2	2	2	0
**Adul’s characteristics**						
High school or GED^c^ (%)	38	38	39	37	37	6
Adult never married (%)	67	70	66	69	67	3
Adult <18 at birth of first child (%)	30	30	29	33	30	4
Adult working for pay (%)	22	20	25	20	21	3
Family size ≤ 2 (%)	9	7	9	9	10	0
Family size = 3 (%)	25	26	23	27	26	0
Family size ≥ 4 (%)	26	23	28	24	27	0
**Adul’s reasons for moving**						
Felt unsafe at night (%)	50	52	50	48	51	0
Dissatisfied with neighborhood (%)	46	45	47	44	47	1
Schools (%)	54	50	57	52	54	3
Drugs/crime (%)	76	77	74	74	79	2
Household had Section 8 before (%)	36	41	34	43	33	1
Moved 3 + times previously (%)	11	16	9	13	10	1
Household received AFDC^d^ (%)	82	81	82	82	82	0
Neighborhood poverty, mean (SD)	0.55 (0.00)	0.56 (0.01)	0.56 (0.00)	0.55 (0.01)	0.55 (0.01)	2
**Intermediate confounder**						
Moved with the voucher (%)	37	0	56	0	54	0
**Mediators**						
*Neighborhood affluence*						
Share time in tract with < 20% poverty, mean (SD)	0.24 (0.01)	0.14 (0.01)	0.28 (0.01)	0.12 (0.01)	0.30 (0.01)	0
Share college graduates, mean (SD)	0.17 (0.002)	0.16 (0.004)	0.18 (0.003)	0.15 (0.005)	0.19 (0.003)	0
*Neighborhood racial/ethnic composition*						
Share Black, mean (SD)	0.51 (0.01)	0.53 (0.01)	0.51 (0.01)	0.51 (0.01)	0.50 (0.01)	0
Share Hispanic, mean (SD)	0.34 (0.01)	0.34 (0.01)	0.33 (0.01)	0.37 (0.01)	0.33 (0.01)	0
*Residential instability*						
Number of moves, mean (SD)	2.75 (2.02)	2.31 (0.09)	2.95 (0.07)	2.32 (0.09)	2.98 (0.06)	0
**Outcomes**						
Past-year mood disorder (%)	9	6	7	15	10	0
Past-year externalizing disorder (%)	18	16	19	20	18	0
Past-year PTSD (%)	4	2	4	6	5	0
Past-year MDD (%)	6	5	4	9	7	0

a Survey weighted, complete case dataset.

b n = unweighted frequency rounded according to Census rules

c Graduate equivalency diploma.

d Aid to Families with Dependent Children.

All results were approved for release by the U.S. Census Bureau, authorization number CBDRB-FY24-CES018-008, CBDRB-FY24-0355, CBDRB-FY25-0191, and CBDRB-FY25-0304

**Table 2:** Adjusted estimates^a^ of the effect of voucher receipt on mediators and outcome in the MTO in Boston, Chicago, New York, and Los Angeles, 1994–2010.

	Boys		Girls	
Mediator/Outcome	Estimate^b^	95% CI	Estimate^b^	95% CI
**Mediators**				
Duration in < 20% poverty	0.20	0.15, 0.25	0.24	0.20, 0.29
College graduates (%)	0.03	0.02, 0.04	0.04	0.03, 0.05
Black (%)	−0.02	−0.04, 0.00	−0.02	−0.04, 0.00
Hispanic (%)	−0.01	−0.03, 0.00	−0.03	−0.05, −0.02
Number of moves	0.63	0.43, 0.84	0.62	0.43, 0.82
**Past-year DSM Outcomes**				
Mood disorder	0.01	−0.01, 0.04	−0.05	−0.08, −0.01
MDD	0.00	−0.02, 0.02	−0.02	−0.05, 0.00
Externalizing disorder	0.02	−0.02, 0.06	−0.03	−0.07, 0.01
PTSD	0.02	0.01, 0.03	−0.01	−0.04, 0.01

a Adjusted for study site, survey weighted and combined across 10 multiply imputed datasets.

b Risk difference.

All results were approved for release by the U.S. Census Bureau, authorization number CBDRB-FY24-CES018–008, CBDRB-FY24–0355, CBDRB-FY25–0191, and CBDRB-FY25–0304.

**Table 3:** Natural direct and indirect effect^a^ of voucher receipt on the risk of mental health outcomes stratified by sex in the MTO in Boston, Chicago, New York, and Los Angeles among adolescents (10 to 19 years), 1994–2010^b^

Sex	Outcome	Effect type	Estimate^b^	95% CI
Boys	Mood disorder	Direct	0.00	−0.03, 0.03
		Indirect	0.02	0.00, 0.03
	MDD	Direct	0.00	−0.03, 0.02
		Indirect	0.01	0.00, 0.02
	Externalizing disorder	Direct	0.05	−0.02, 0.13
		Indirect	−0.03	−0.09, 0.03
	PTSD	Direct	0.02	0.00, 0.05
		Indirect	0.00	−0.02, 0.02
Girls	Mood disorder	Direct	−0.03	−0.08, 0.01
		Indirect	−0.01	−0.04, 0.02
	MDD	Direct	−0.01	−0.05, 0.02
		Indirect	−0.01	−0.03, 0.01
	Externalizing disorder	Direct	−0.01	−0.06, 0.04
		Indirect	−0.02	−0.05, 0.02
	PTSD	Direct	−0.01	−0.04, 0.02
		Indirect	0.00	−0.02, 0.02

a Bundle of mediators included proportion of time spent in low-poverty census tracts, share of college graduates, Black, and Hispanic residents.

b Survey weighted and combined across 10 multiply imputed datasets.

c Risk difference.

All results were approved for release by the U.S. Census Bureau, authorization number CBDRB-FY24-CES018-008, CBDRB-FY24-0355, CBDRB FY25 0191, and CBDRB-FY25-0304.

**Table 4:** Natural direct and indirect effect^a^ of voucher receipt on the risk of mental health outcomes stratified by sex in the MTO in Boston, Chicago, New York, and Los Angeles among adolescents (10 to 19 years), 1994–2010^b^.

Sex	Outcome	Effect type	Estimate^b^	95% CI
**Boys**	Mood disorder	Direct	0.00	−0.03, 0.03
		Indirect	0.02	0.00, 0.03
	MDD	Direct	0.00	−0.03, 0.02
		Indirect	0.01	0.00, 0.02
	Externalizing disorder	Direct	0.05	−0.02, 0.12
		Indirect	−0.02	−0.09, 0.04
	PTSD	Direct	0.02	0.00, 0.04
		Indirect	0.00	−0.01, 0.02
**Girls**	Mood disorder	Direct	−0.03	−0.08, 0.01
		Indirect	−0.01	−0.04, 0.02
	MDD	Direct	−0.02	−0.05, 0.02
		Indirect	−0.01	−0.03, 0.01
	Externalizing disorder	Direct	−0.01	−0.06, 0.04
		Indirect	−0.01	−0.05, 0.02
	PTSD	Direct	−0.01	−0.04, 0.02
		Indirect	0.000	−0.02, 0.02

a Bundle of mediators included proportion of time spent in low-poverty census tracts, share of college graduates, Black, and Hispanic residents.

b Survey weighted and combined across 10 multiply imputed datasets.

c Risk difference.

All results were approved for release by the U.S. Census Bureau, authorization number CBDRB-FY24-CES018-008, CBDRB-FY24-0355, CBDRB FY25 0191, and CBDRB-FY25-0304.

## Data Availability

Data can be accessed upon request to the U.S. Census Bureau following the instructions outlined in: https://www.census.gov/abouat/adrm/linkage/projects/HUDmtofos.html
